# C-852-07. The EMBER Score: Identifying Patients at Risk for Early Renal Replacement After Severe Burns

**DOI:** 10.1093/jbcr/irag033.067

**Published:** 2026-04-06

**Authors:** Ryan D Rosen, Ryan T Davis, Alfredo E Munoz-Laroche, Silvia Aluia, Meredith Hazelrigg, Basel Mhaimeed, Heather Dolman, Samantha Tarras, Michael T White

**Affiliations:** Detroit Receiving Hospital Burn Center, Detroit, MI; Wayne State University School of Medicine, Children's Hospital of Michigan Burn Center, Detroit, MI; Detroit Receiving Hospital Burn Center, Detroit, MI; Detroit Receiving Hospital Burn Center, Detroit, MI; Detroit Receiving Hospital Burn Center, Detroit, MI; Wayne State University School of Medicine, Detroit, MI; Detroit Receiving Hospital Burn Center, Detroit, MI; Indiana University Health, Indianapolis, IN; Detroit Receiving Hospital Burn Center, Detroit, MI

## Abstract

**Introduction:**

Acute kidney injury (AKI) occurs in 20–40% of burn patients and significantly increases mortality. Acute renal failure requiring renal replacement therapy (RRT) is less frequent (2-8%) but carries very high mortality (40-80%). Known risk factors include patient (age, comorbidities) and injury characteristics (total body surface area [TBSA], inhalation injury, failed resuscitation). Predictive models have been proposed to predict AKI after burn injury, but to our knowledge, no model to specifically predict RRT has been developed. We aim to identify independent risk factors and create a model to predict early RRT (≤14 days) after burns.

**Methods:**

A retrospective study evaluated admissions to the Burn ICU at a single American Burn Association-verified burn center from January 2013 to December 2022. Patients admitted for <72 hrs or with pre-existing renal disease were excluded. Derivation and validation cohorts were defined using odd and even years. Multivariable logistic regression identified independent predictors of RRT, which were incorporated into the EMBER (Evaluation Model for Burn-associated Early Renal failure) Score. Validation was performed using the area under the receiver-operating-characteristic (AUROC) curves. Significance was set at *p*<.05.

**Results:**

Among 687 included patients. 60 (8.7%) required RRT, median initiation was HD5 (IQR 3-10). Mortality was higher in patients requiring RRT (71.7% vs 9.9%, OR 23.1, *p*<.001). The derivation cohort (n = 315; 23 [7.3%] RRT) yielded nine predictors of RRT within 14 days. Four variables (overall TBSA ≥40% or full-thickness TBSA ≥20%, inhalation injury, rhabdomyolysis, and bicarbonate <17.5 or lactate >4) comprised the admission component. The five delayed variables (failure of resuscitation, oliguria, and HD3 hyperphosphatemia, serum creatinine ≥1.5, and lactate ≥2.75) completed the EMBER Score. In the validation cohort (n = 372; 37 [9.9%] RRT), the admission component achieved AUROC 0.872 (95% CI 0.804–0.940), while the combined EMBER Score achieved AUROC 0.958 (0.926–0.990). The maximum score is 13; observed RRT rates were 5.9%, 33.3%, 83.3% and 100% with scores of 4, 7, 10, and 13, respectively.

**Conclusions:**

The EMBER score is a novel tool to predict early renal failure after burns. While it shows promise at stratifying patient risk for RRT, refinement and prospective validation are necessary before clinical adoption.

**Applicability of Research to Practice:**

Prospective validation of the EMBER Score could enable earlier recognition and clinical intervention for burn patients at risk of renal failure. It also provides a framework to define high-risk cohorts for future studies on early RRT initiation.

**Funding for the study:**

N/A.

